# Correct anemia or prevent acute myocardial infarction in patients on maintenance hemodialysis?

**DOI:** 10.1080/0886022X.2019.1662441

**Published:** 2019-09-13

**Authors:** Yu Zhao, Wenyun Wang, Zhichun Dong, Jie Feng, Shijie Ma

**Affiliations:** a Department of Pediatric Surgery, Second Hospital of Lanzhou University, Lanzhou, PR China;; b Department of Urology, Second Hospital of Lanzhou University, Lanzhou, PR China;; c Department of Gastroenterology, Second Hospital of Lanzhou University, Lanzhou, PR China;; d Department of Medicine, Northwest Minzu University, Lanzhou, PR China

Dear Sir,

After careful reading through the article called ‘High sensitivity Troponin-I levels in asymptomatic hemodialysis patients’ by Tarapan et al. [1]. Cardiac troponin-I is a well-known crucial parameter in diagnosis of acute myocardial infarction (AMI). The article showed that the high-sensitivity troponin I (hsTnI) levels in hemodialysis (HD) group were higher than non- chronic kidney disease (CKD) group (*p* < .001). The hsTnI levels reduced after HD process from 54.3 ng/L (20.6–152.7) in pre-HD to 27.1 ng/L (12.3–91.4) in post-HD (*p*  =  .015). About 25% of HD patients had increment of hsTnI after HD. Subgroup analysis displayed that the hemoglobin (Hb) higher than 11 g/dL (*p* = .013) was identified as the independent factors associated with hsTnI elevation in HD patients with post-dialysis. I pay special attention to the results of this research because it caused me some confusion. First, as we all know that renal anemia is one of the most important complications in patients on maintenance hemodialysis (MHD) and underlies a range of symptoms including fatigue, depression, reduced exercise tolerance, and dyspnea. It is associated with morbidity and mortality related to cardiovascular disease and increased risk of hospitalization in addition to a reduction in exercise capacity, and quality of life. The KDIGO guidelines, European Best Practices Guideline, and The National Kidney Foundation Kidney Disease Outcome Quality Initiative guidelines recommend a target Hb level of 11–12 g/dL in patients on MHD [2–4]. Pisoni et al. reported that patient mortality and hospitalization risks were shown to decrease by 10–12% for every 1 g/dL increase in mean facility-level Hb [5].

Second, a meta-analysis by Ye et al. [6] showed that no significant difference was found in all-cause mortality in the fixed effects model (RR 1.09, 95% CI 0.93–1.27; *p* = .30), cardiovascular events (RR 0.77, 95% CI 0.31–1.92; *p* = .58), infectious diseases (RR 0.69, 95% CI 0.24–1.96; *p* = .49) and transfusion (RR 0.92, 95% CI 0.42–1.99; *p* = .82) in the random-effects model between the higher Hb target group and the lower Hb target group. Therefore, as a clinician, in the face of MHD patients, how do we weigh the balance between anemia and myocardial infarction? Tarapan et al. [1] research results are very meaningful. The association between Hb and hsTnI in MHD patients is complicated and urgently needs more studies to confirm.

Finally, I found some problems with the percentages of gender, diabetes mellitus, and hypertension are incorrectly calculated in Table 2 [1]. Therefore, the accuracy of the data has yet to be further verified by the author.

## References

[1] Tarapan T, Musikatavorn K, Phairatwet P, et al. High sensitivity Troponin-I levels in asymptomatic hemodialysis patients. Ren Fail. 2019;41(1):393–400. 31132904 10.1080/0886022X.2019.1603110 PMC6542185

[2] Locatelli F, Aljama P, Barany P, et al. Revised European best practice guidelines for the management of anaemia in patients with chronic renal failure. Nephrol Dial Transplant. 2004;19(Suppl 2):ii1-47. 10.1093/ndt/gfh1032 15206425

[3] KDOQI KDOQI Clinical Practice Guideline and Clinical Practice Recommendations for anemia in chronic kidney disease: 2007 update of hemoglobin target. Am J Kidney Dis. 2007;50(3):471–530. 17720528 10.1053/j.ajkd.2007.06.008

[4] KDIGO work Group Chapter 1: diagnosis and evaluation of anemia in CKD. Kidney Int Suppl (2011). 2012;2(4):288–291. 25018948 10.1038/kisup.2012.33 PMC4089684

[5] Pisoni RL, Bragg-Gresham JL, Young EW, et al. Anemia management and outcomes from 12 countries in the Dialysis Outcomes and Practice Patterns Study (DOPPS). Am J Kidney Dis. 2004;44(1):94–111. 15211443 10.1053/j.ajkd.2004.03.023

[6] Ye Y, Liu H, Chen Y, et al. Hemoglobin targets for the anemia in patients with dialysis-dependent chronic kidney disease: a meta-analysis of randomized, controlled trials. Ren Fail. 2018;40(1):671–679. 30741617 10.1080/0886022X.2018.1532909 PMC6282462

